# Screening of *brain-derived neurotrophic factor (BDNF)* single nucleotide polymorphisms and plasma BDNF levels among Malaysian major depressive disorder patients

**DOI:** 10.1371/journal.pone.0211241

**Published:** 2019-01-24

**Authors:** Asraa Faris Aldoghachi, Yin Sim Tor, Siti Zubaidah Redzun, Khairul Aiman Bin Lokman, Nurul Asyikin Abdul Razaq, Aishah Farhana Shahbudin, Ibrahim Mohamed Badamasi, Pike-See Cheah, Johnson Stanslas, Abhi Veerakumarasivam, Rozita Rosli, Normala Ibrahim, Munn Sann Lye, King-Hwa Ling

**Affiliations:** 1 Department of Biomedical Sciences, Universiti Putra Malaysia, Serdang, Selangor, Malaysia; 2 Department of Community Health, Universiti Putra Malaysia, Serdang, Selangor, Malaysia; 3 Department of Psychiatry, Universiti Putra Malaysia, Serdang, Selangor, Malaysia; 4 Department of Medicine, Universiti Putra Malaysia, Serdang, Selangor, Malaysia; 5 Department of Human Anatomy, University Putra Malaysia, Serdang, Selangor, Malaysia; 6 Department of Biological Sciences, School of Science and Technology, Sunway university, Subang Jaya, Selangor, Malaysia; Chiba Daigaku, JAPAN

## Abstract

**Background:**

*Brain-derived neurotrophic factor (BDNF*) is a neurotrophin found in abundance in brain regions such as the hippocampus, cortex, cerebellum and basal forebrain. It has been associated with the risk of susceptibility to major depressive disorder (MDD). This study aimed to determine the association of three *BDNF* variants (rs6265, rs1048218 and rs1048220) with Malaysian MDD patients.

**Methods:**

The correlation of these variants to the plasma BDNF level among Malaysian MDD patients was assessed. A total of 300 cases and 300 matched controls recruited from four public hospitals within the Klang Valley of Selangor State, Malaysia and matched for age, sex and ethnicity were screened for *BDNF* rs6265, rs1048218 and rs1048220 using high resolution melting (HRM).

**Findings:**

*BDNF* rs1048218 and *BDNF* rs1048220 were monomorphic and were excluded from further analysis. The distribution of the alleles and genotypes for *BDNF* rs6265 was in Hardy-Weinberg equilibrium for the controls (*p* = 0.13) but was in Hardy Weinberg disequilibrium for the cases (*p* = 0.011). Findings from this study indicated that having *BDNF* rs6265 in the Malaysian population increase the odds of developing MDD by 2.05 folds (95% CI = 1.48–3.65). Plasma from 206 cases and 206 controls were randomly selected to measure the BDNF level using enzyme-linked immunosorbent assay (ELISA). A significant decrease in the plasma BDNF level of the cases as compared to controls (*p*<0.0001) was observed. However, there was no evidence of the effect of the rs6265 genotypes on the BDNF level indicating a possible role of other factors in modulating the BDNF level that warrants further investigation.

**Conclusion:**

The study indicated that having the *BDNF* rs6265 allele (A) increase the risk of developing MDD in the Malaysian population suggesting a possible role of BDNF in the etiology of the disorder.

## Introduction

Major depressive disorder (MDD) is one of the most common mental illnesses worldwide affecting an individual’s feelings, thoughts and behaviour. It results from an interaction of both genetic and environmental factors. The symptoms of major depressive disorder usually last for two or more constitutive weeks. The symptoms are characterised by having a persistent form of sadness and loss of interest in once pleasurable activities and several other physical and cognitive symptoms such as inability to concentrate, changes in sleep and eating habits, physical inactivity, suicidal thoughts and suicidality [1]. Currently, MDD is a major cause to the global burden of disease affecting more than 300 million individuals globally [2]. It is the third most disabling disorder worldwide and is expected to be the first by the year 2030 [3]. In Malaysia, it is one of the most common reported mental illnesses with about 29% of the Malaysians suffering from depression in the year 2015 [4].

A number of studies indicate the involvement of the brain derived neurotrophic factor (BDNF) in the aetiology of the disorder [5–7]. BDNF is a neurotrophin that plays a crucial role in neuronal development, differentiation, survival and synaptic plasticity [8–11]. One of the most reported and most functional single nucleotide polymorphisms in the *BDNF* gene is rs6265. *BDNF* rs6265 has been extensively studied in patients with MDD, however, the results were controversial, depicting outcome differences in populations and ethnicities among the groups studied [12–16]. The Met allele of *BDNF* rs6265 was shown to alter the intracellular trafficking and secretion of the mature BDNF protein as compared to the Val allele [17–19]. Additionally, several studies have reported lower plasma BDNF levels in patients with MDD [5,20,21]. Low levels of the BDNF protein was also found in the post-mortem amygdala [22], anterior cingulate cortex [23], prefrontal cortex [24] and hippocampus [24,25] of depressed patients.

Other SNPs of interest in this study were *BDNF* rs1048218 and rs1048220. Despite the availability of literature associating the two SNPs to Alzheimer’s disease [26,27], the literature regarding the functional and physiological role of these SNPs has been scarce. *BDNF* rs1048220 is situated at an important protease cleavage site and was shown to inhibit the cleavage of pro-BDNF of cultured hippocampal neurons [28]. To date, there has been no available study screening for associated risks of *BDNF* rs1048218 and rs1048220 SNPs among patients with MDD. Also, to the best of our knowledge, there is no study on the association of *BDNF* rs6265, rs1048218, rs1048220 and BDNF level with MDD in the Malaysian population. In this study, we screened for *BDNF* rs6265, rs1048218 and rs1048220 in 600 matched Malaysian cases and controls and the effect of the genotypes on the level of the mature BDNF protein in the plasma.

## Methodology

### Study design

Ethical approval to conduct the study was obtained from the Medical Research and Ethics Committee of National Institutes of Health Malaysia (reference number: NMRR-14-688-19696 (IIR)). Ethical approval to conduct the study was obtained from the Medical Research and Ethics Committee of National Institutes of Health Malaysia (reference number: NMRR-14-688-19696 (IIR)). The approval includes procedures involved in the recruitment of subjects, obtaining written consent, performing semi-assisted questionnaires, samples procurement and usage. All procedures complied with Helsinki’s declaration. A total of 400 case and 400 controls matched by age, gender and ethnicity were recruited from four public hospitals within the Klang Valley of Selangor State, Malaysia from the year 2014–2018. All the subjects were adults, mentally competent and were clearly informed about the study procedures and the study outcomes. The study subjects were aware that joining the study was a voluntary decision and were explained about the study both verbally and by providing them with written information; a 9 page consent in the English and the local Malay language regarding the title and purpose of research, research background, study design, eligibility of joining the study, study procedures, subject responsibilities, potential benefits and the possible risks of joining the study, risks of SSRI intake and risk of blood draws, right to ask questions or withdraw from the study and the use of study results and confidentiality. For older subjects, prior to obtaining the consent from the subjects, the study details were explained to them as well as to the person accompanying them. The subjects were free to ask questions regarding the study and were given the freedom to withdraw from the study at any time if they wanted to.

#### Definition of cases

The cases were outpatients within the age group of 18–65 diagnosed with major depressive disorder (MDD) by a certified psychiatrist based on the Diagnostic and Statistical Manual of Mental Disorders, Fifth edition (DSMV) and confirmed by the by Mini International Neuropsychiatric Interview (MINI) for MDD. Only cases diagnosed by the psychiatrist as MDD with single episode or recurrence and without psychotic features were recruited in this study. Both new and follow up patients (on medication) were allowed to join the study. Furthermore, only cases with a history of MDD for two years or less prior to screening were recruited in this study. Subjects with risk of committing suicide and with a current DSMV diagnosis of other neuropsychiatric illness such as generalized anxiety disorder, obsessive compulsive disorder, post-traumatic stress, dementia, schizophrenia or other psychotic disorder, bipolar I/II disorder and anxiety disorders were excluded.

#### Definition of controls

The controls were adult outpatients attending the orthopaedic, ophthalmology and ear, nose and throat (ENT) clinics of four public hospitals within the Klang Valley of Selangor State. Controls were also recruited from the staff and students of Universiti Putra Malaysia. Controls were unrelated to the cases and were matched for age, gender and ethnicity to the cases in a 1:1 ratio. Only controls with no history of other psychiatric disorders were recruited.

### Overview of the study

Consented subjects were asked to filled a questionnaire on demographic data (such as age, gender, ethnicity, religion, marital status, citizenship, job status, total household income per month and education level), clinical characteristics (such as the acquisition of any chronic disease, and duration of symptom and the presence of a family history of mental health issues), list of threatening events (a list of 12 yes or no questions on the experience of life threatening events in 6 months prior to the interview), temperament and personality questionnaire (a list of 109 questions on the general feelings of the participants over the years), semiquantitative food frequency questionnaire (participants were asked to fill in the frequency of cereal products, meat products, fish products, sea food, milk, dairy products, vegetables, fruits, drinks and confectioners consumed on a daily basis, for 2–3 times per week, weekly, monthly and annually and were asked to provide the number of servings), supplement intake (a list of yes/ no questions on the consumption of 16 food supplement and 10 vitamin/mineral supplement and the frequency of the consumption), lifestyle questionnaire (on smoking and the duration and alcohol consumption, the type of drinks usually consumed and the frequency), global physical activity questionnaire (on the intensity and duration of the physical activity during working and day to day activities), blood pressure level and anthropometry measurement (weight, height, waist and hip circumference, body fat, BMI and waist hip ratio). For participants who found it difficult to fill out the questionnaire, the process was completed via interview. Only data regarding the demographics, clinical characteristics and life style were used for this study whereas the rest of the information were used for a different study. Subjects were also asked to consent to supply 4ml of venous blood sample. The collected blood was centrifuged at 4,000 rpm for 10 minutes at room temperature to obtain the buffy coat and plasma. All the samples were then stored at -20°C for future use.

#### DNA extraction and DNA integrity

The genomic DNA was extracted from the buffy coat of anti-coagulated blood samples using QIAamp DNA Mini Kit (QIAGEN) following the manufacturer’s protocol. The quality of the DNA was determined using NanoVue Plus UV spectrophotometer (GE Healthcare). Only samples with A260/A280 absorbance of 1.7–1.9 were used and regarded as high-quality samples. The extracted DNA were electrophoresed using 1% (w/v) agarose gel for 40 minutes at 100V. The bands were viewed using G:BOX BioImaging System (Syngene). All the extracted DNA were stored at -20°C until further use.

#### Screening of the SNPs

Primers for the screening of *BDNF* rs6265, rs1048218, rs1048220 were designed using Primer3Plus (Table 1) [29]. High resolution melting was carried out using LightCycler 480 (Roche, Switzerland) in a volume of 10μl consisting of 1X LightCycler 480 High Resolution Melting Master (Roche, Switzerland), 15ng of genomic DNA and 0.35μmol forward and reverse primers, 2mM MgCl_2_ and PCR-grade water. The conditions for the thermal cycling were as follows: initial denaturation at 95°C for 10 min followed by 45 cycles of amplification at 95°C for 10s and annealing at 60°C for 15s and a final extension at 72°C for 10s. Subsequently, amplicons were subjected to HRM analysis followed by melting analysis in the same machine using a temperature range of 65°C to 95°C with 25 acquisitions per every 1°C increment. To obtain the best discrimination, samples with different melting profiles denoting the different genotypes were randomly selected from the first run and were validated by sequencing and were included in each run to serve as references. The HRM data were analysed using the Light Cycler release 1.5.1.62SP3 software. The normalized melting curves and the temperature shifted differential plots were obtained from the gene scanning module of the software to determine genotype for each sample based on the profiles of samples validated by sequencing.

**Table 1:** The criteria of the designed primers for high resolution melting assays.

*BDNF* variant	Forward (F) / Reverse (R) primer (5’→3’) / Tm (°C)	Amplicon size (bp)	GC Content (%)
rs6265*	F: CTTGACATCATTGGCTGACACT (60)	146	45
	R:GCTCCAAAGGCACTTGACTACT (60)		50
rs1048220	F: CCTTTGGAGCCTCCTCTTCT (56.4)	150	55
	R: CGCCGTTACCCACTCACTAA (56.9)		55
rs1048218	F: GCTTGACATCATTGGCTGAC (57.4)	157	50
	R:AGAAGAGGAGGCTCCAAAGG (58.4)		55

*The parameters were based on Faris et al., 2018 [29].

#### Sequencing

HRM products for approximately 10% of the samples were purified using High Pure PCR purification kit, (Roche, Switzerland) following the manufacturer’s instructions. Purified products with high purity (absorbance 260/280 of 1.6–2.0) and integrity (single clear band by agarose gel analysis) were sent for Sanger sequencing using services provided by First BASE Laboratories Sdn Bhd, Malaysia. The assembly of the sequences and the generation of the contigs was performed by using SNAPGENE software (GSL Biotech; available at snapgene.com). Phred score of >20 was used as a cut-off for good quality sequences.

#### Measurement of BDNF level using ELISA

Sandwich enzyme linked immunosorbent assay was carried out to measure the level of the BDNF protein in the plasma of 206 matched MDD cases and 206 controls. The ELISA was carried out using BDNF Emax ImmunoAssay kit (Promega, United States) following the manufacturer’s protocol without the acid treatment step on plasma samples. Firstly, the 96 well plate (Nunc MaxiSorp, USA) was coated with 100 μL anti-BDNF monoclonal antibody by overnight incubation at 4°C without shaking. To prevent the unspecific binding, plasma samples (1:16 dilution) and the standards (1:2000 dilutions) were added to the 96-well plate (100 μl each) and incubated at room temperature for 2 hours on shaker. The samples were then incubated with anti-human BDNF polyclonal antibody for 2 hours at room temperature on shaker. The plate was then incubated with anti-IgY HRP at room temperature for 1 hour. 3,3’,5,5’-tetramethylbenzidine was then added and incubated for 10 minutes to start the colour reaction. The reaction was terminated by the addition of 1N hydrochloric acid and the absorbance at 450 nm was measured instantly using the microplate reader (VersaMax Microplate Reader). The readings were calculated from the extrapolated equation of the standard curve.

#### Statistical analysis

Chi-square test was performed using SPSS to determine if there is any significant difference of genotype or allele frequencies among the cases and controls and to test for the significance in the distribution of genotypes among the varying ages, genders and ethnicities of the cases and controls. Following that, the distribution of the genotype frequencies among the cases and controls was determined using SNPStats. Chi-square was also used to measure the deviation of the genotypes from Hardy-Weinberg equilibrium (HWE). SNPStats was used to analyse the following inheritance models: the codominant model, dominant model, recessive model and the overdominant model. Multivariable logistic regression using SPSS was used to study the association of genotype to MDD and determine adjusted odds ratio and 95% CI, adjusting for age, gender, ethnicity, religion, job, income, education level, alcohol consumption, family history of neuropsychiatric disorders, depression, anxiety, schizophrenia, stroke, chronic disease, heart disease, diabetes, hypertension, arthritis, cancer and asthma. Benjamini-Hochberg method for adjustment of false discovery rate (FDR) for haplotype analysis was performed using web-based tool (https://www.sdmproject.com/utilities/?show=FDR). The data obtained from ELISA were analysed using GraphPad prism. The comparison between the cases and controls was performed using Wilcoxon signed rank test for non-parametric data. Kruskal–Wallis test was used to measure the significance among the three genotypes for a non-normally distributed result. Significance was set at *p*<0.05.

## Results

Out of the 800 subjects approached for this study, only 300 cases and 300 matched controls met the criteria to join the study. The remaining subjects were rejected as they were diagnosed with anxiety or other neuropsychiatric disorders, refused to consent for blood withdrawal and/or were uncontactable after initial agreement to participate in the study. The socio-demographic information for all the participants included in the study are listed in Table 2.

**Table 2:** The sociodemographic data for the cases and controls.

Parameters	Categories	Cases	Controls
**Age group (years)**	18–25	52 (17.3%)	55 (18.3%)
	26–35	76 (25.3%)	89 (29.7%)
	36–45	83 (27.7%)	62 (20.7%)
	46–55	48 (16%)	55 (18.3%)
	56–65	41(13.7%)	39 (13%)
**Gender**	Male	97 (32.3%)	97 (32.3%)
	Female	203(67.7%)	203 (67.7%)
**Race**	Chinese	90 (30.0%)	90 (30.0%)
	Indian	58 (19.3%)	58 (19.3%)
	Malay	149(49.7%)	149(49.7%)
	Others	3 (1%)	3 (1%)
**Religion**	Buddhist	66 (22.0%)	68 (22.7%)
	Christian	26 (8.7%)	26 (8.7%)
	Hindu	46 (15.3%)	46 (15.3%)
	Muslim	156 (52.0%)	152 (50.7%)
	Others	6 (2.0%)	8 (2.7%)
**Marital status**	Married	141 (47%)	171 (57%)
	Divorced	20 (6.7%)	7 (2.3%)
	Single	118 (39.3%)	112 (37.3%)
	Widowed	16 (5.3%)	10 (3.3%)
	Others	5 (1.7%)	0 (%)
**Job status**	Student	41 (13.7%)	64 (21.3%)
	Semi-government	4 (1.3%)	18 (6.0%)
	Government	43 (14.3%)	103 (34.3%)
	Private	85 (28.3%)	66(22.0%)
	Retired	29 (9.7%)	23 (7.7%)
	Others	98 (32.7%)	26 (8.7%)
**Income level (RM)**	<1000	87 (29%)	28 (9.3%)
	1001–2000	65(21.7%)	57 (19.0%)
	2001–3000	64 (21.3%)	74 (24.7%)
	3001–4000	25 (8.4%)	51(17.0%)
	>4000	59 (19.7%)	90 (30.0%)
**Education level**	Primary/secondary	147 (49.0%)	89 (29.7%)
	Diploma	45(15.0%)	82 (27.3%)
	Degree/postgraduate	83 (27.7%)	102 (34.0%)
	Certificate	25 (8.3%)	27 (9.0%)
**Alcohol consumption**	Yes	58 (19.3%)	73 (24.3%)
	No	242 (80.7%)	227 (75.7%)
**Smoking status**	Yes	36 (12.0%)	60 (20.0%)
	No	251(83.7%)	221 (73.7%)
	Given up	13 (4.3%)	19 (6.3%)
**Family history of neuropsychiatric disorder**	Yes	83 (27.7%)	14 (4.7%)
	No	217 (72.3%)	286 (95.3%)
**Family history of depression**	Yes	59 (19.7%)	4 (1.3%)
	No	241 (80.3%)	296 (98.7%)
**Family history of anxiety**	Yes	10 (3.3%)	1 (0.3%)
	No	290 (96.7%)	299 (99.7%)
**Family history of schizophrenia**	Yes	14 (4.7%)	5 (1.7%)
	No	286 (95.3%)	295 (98.3%)
**History of stroke**	Yes	2 (.7%)	1 (0.3%)
	No	298 (99.3%)	299 (99.7%)
**History of chronic disease**	Yes	106 (35.3%)	94 (31.3%)
	No	194 (64.7%)	206 (68.7%)
**History of heart disease**	Yes	7 (2.3%)	3 (1%)
	No	293 (97.7%)	297 (99%)
**History of diabetes**	Yes	28 (9.3%)	23 (7.7%)
	No	272 (90.7%)	277 (92.3%)
**History of hypertension**	Yes	48 (16.0%)	37 (12.3%)
	No	252 (84%)	263 (87.7%)
**History of arthritis**	Yes	8 (2.7%)	7 (2.3%)
	No	292 (97.3%)	293 (97.7%)
**History of cancer**	Yes	0 (0%)	4 (1.3%)
	No	300 (100%)	296 (98.7%)
**History of asthma**	Yes	24 (8%)	20 (6.7%)
	No	276 (92%)	280 (93.3%)

27.7% of the cases in this study were within the age group of 36–45 and were females (67.7%). Cases with the Malay ethnicity made up most of the cases (49.7%) as compared to the other ethnicities studied. Most of the cases were married (47%), with primary education (49%) and with low income level (<RM1000; 29%). Majority cases had no history of neuropsychiatric disorders and no history of chronic disorders.

Majority of the cases recruited in the study were patients attending their follow-up examination at the psychiatric clinic. Therefore, 92.5% of the cases were already on selective serotonin reuptake inhibitor (SSRI) medication (Table 3). Most of them were given fluovoxamine (48%) whereas nearly equal number of cases were prescribed sertraline (21%) or escitalopram (21%). Only a small proportion of the cases were under fluoxetine (2%) medication. A total of 17 new cases (6%) were not on any medications at the time of sample collection. Unknown medication status was recorded on 5 cases in the study.

**Table 3:** Prescribed medications at the time of sample collection.

SSRI*	Number of cases	%
Fluovoxamine	143	47.7
Sertraline	64	21.3
Escitalopram	62	20.7
Fluoxetine	7	2.3
Others	2	0.7
No medications	17	5.7
Unknown	5	1.7
Total	300	100

* Selective serotonin reuptake inhibitors

### Genotyping and validation of *BDNF* SNPs

**Fig 1:**
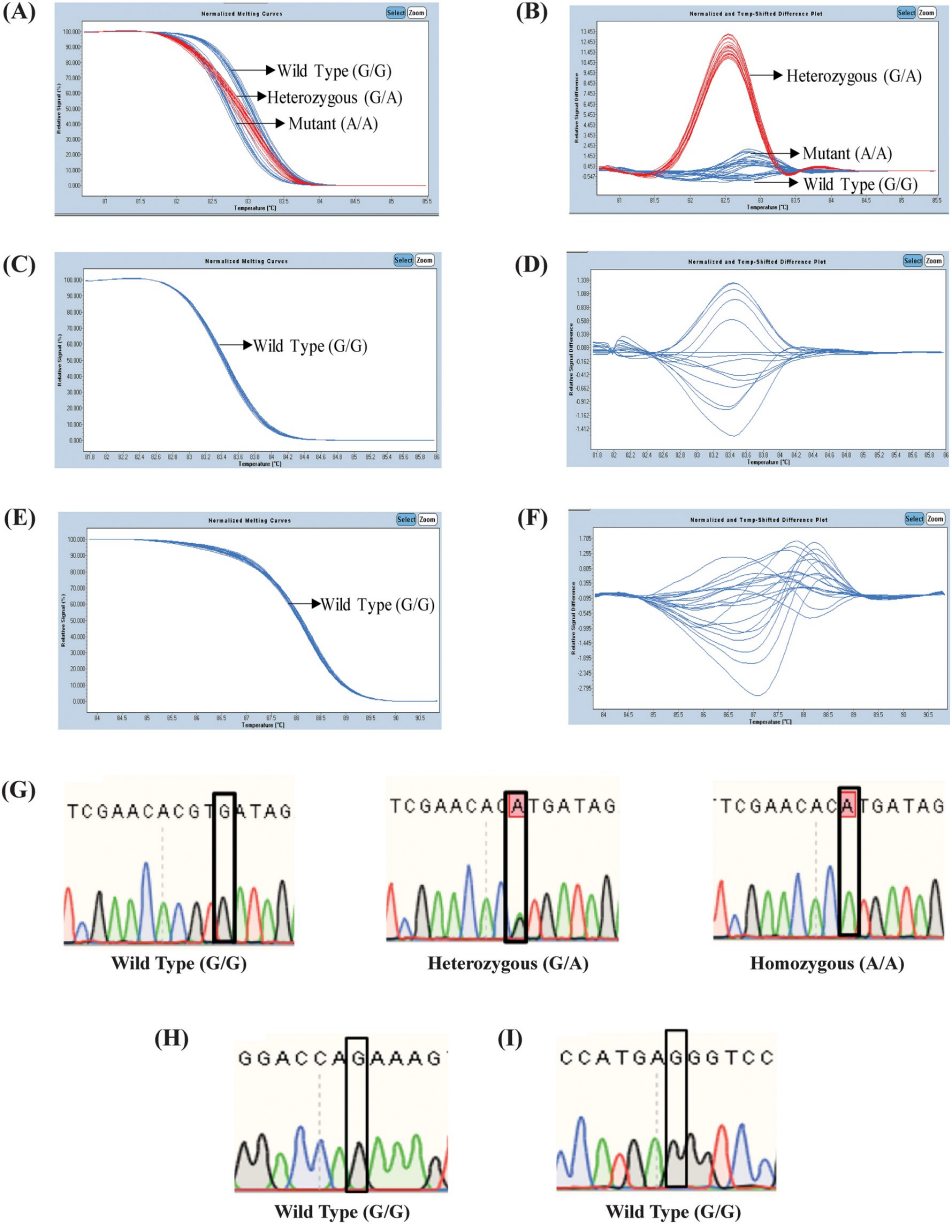
High-resolution melting analysis. A representative result of the normalized melting curves for (A) each genotype G/G, A/A and G/A *BDNF* rs6265 (C) *BDNF* rs1048218 and (E) *BDNF* rs1048220 and temperature shifted difference plot for (B) the three genotypes G/G, A/A and G/A of *BDNF* rs6265 (D) *BDNF* rs1048218 and (F) *BDNF* rs1048220. (G) Representative results for the G/G, G/A, and A/A genotypes obtained from the sequencing of *BDNF* rs6265. A representative result for the monomorphic genotype obtained from the sequencing of (H) *BDNF* rs1048218 and (I) *BDNF* rs1048220.

A total of 300 cases with MDD and 300 matched healthy controls were screened for *BDNF* rs6265 using HRM. Three distinct peaks were obtained for *BDNF* rs6265 indicating the three genotypes (wild type, heterozygous, recessive). The distinction of the three genotypes was based on differences in the melting temperature and the shape of the melting curve. The wild type and the mutant genotypes were discriminated by changes in the melting temperature, whereas the discrimination of heterozygous genotypes was displayed by a change in the shape of the melting curve (Fig 1A and 1B).

The HRM result showed that 73 cases had wild type genotype (G/G), 100 cases had the mutant variant (A/A), and 127 cases had the heterozygous genotype (G/A). On the other hand, of the screened controls, 97 were genotyped as G/G, 68 were genotyped as A/A and 135 were genotyped as G/A. In contrast, *BDNF* rs1048218 (Fig 1C and 1D) and *BDNF* rs1048220 (Fig 1E and 1F) displayed a monomorphic genotype throughout the screening of 245 cases and 245 controls and both variants were excluded from further analysis.

Out of the 600 screened subjects, a total of 17 G/G (10%), 26 G/A (10%) and 17 A/A (10%) samples were sent for sequencing. The sequencing results were 100% matched with the HRM screening confirming the specificity of the assay (Fig 1G). On the other hand, the sequencing electropherogram of 30 (12%) samples for *BDNF* rs1048218 and 30 (12%) samples for *BDNF* rs1048220 showed a monomorphic nature, which were 100% concurring with the HRM analysis (Fig 1H and 1I).

Upon stratifying the genotypes among the different genders of the cases and controls, a significant difference was obtained among the distribution of genotypes in the female cases as compared to the controls (*p* = 0.007). However, no significant difference was obtained among the male case and control subjects (Table 4).

**Table 4:** The stratification of genotypes among male and female case and control subjects.

Gender	Case*			Control*			*p* value
	Genotype (%)			Genotype (%)			
	Wild type	Mutant	Heterozygous	Wild type	Mutant	Heterozygous	
**Male**	26 (26.8)	29 (29.9)	42 (43.3)	35 (36.1)	25 (25.8)	37 (38.1)	0.379
**Female**	47 (23.2)	71 (35)	85 (41.9)	62 (30.5)	43 (21.2)	98 (48.3)	0.007

* Values in parenthesis are percentages

Additionally, when stratifying the genotypes among the different Malaysian ethnicities of the cases and controls, a significant difference was obtained for the Chinese (*p* = 0.001) and Indian ethnicities (*p* = 0.002) but not for the Malay ethnicity (Table 5). Other rare ethnicities were excluded from the analysis.

**Table 5:** The stratification of genotypes among the ethnicities of the cases and controls.

Ethnicity	Case*			Control*			*p* value
	Genotype (%)			Genotype (%)			
	Wild type	Mutant	Heterozygous	Wild type	Mutant	Heterozygous	
**Malay**	35 (23.8)	35 (23.8)	77 (52.4)	48 (32.4)	41 (27.7)	59 (39.9)	0.087
**Chinese**	14 (15.6)	42 (26.7)	34 (37.8)	26 (28.9)	19 (21.1)	45 (50.0)	0.001
**Indian**	23 (39)	21 (35.6)	15 (25.4)	20 (34.5)	7 (12.1)	31 (53.4)	0.002

* Values in parenthesis are percentages

### The allele and genotype distribution among the cases and controls

Using Chi-square, a significant difference of the frequency of the alleles and genotypes was observed among the cases and controls (*p* = 0.008). From the observed HRM genotypes, the allele and genotype frequencies were obtained. Majority of the cases had the mutant allele A (55%) whereas the rest (46%) had the allele G. On the other hand, for the controls, majority demonstrated the wild type allele G (55%) as compared to the mutant allele A that was only observed by 45% of the participants. From the 300 screened cases, 24% had the wild type genotype (G/G), 42% had the heterozygous genotype (G/A) and 33% had the mutant genotype (A/A). On the other hand, for the 300 screened controls, 32% had the wild type genotype, 45% had the heterozygous genotype whereas the remaining 23% had the mutant genotype. Table 6 summarizes the allele and genotype frequencies among the cases and controls. The distribution of the genotypes for the cases was in Hardy-Weinberg disequilibrium (*p*<0.05) whereby the distribution of the genotypes for the controls was in Hardy-Weinberg equilibrium (*p*>0.05).

**Table 6:** The summary of the distribution of the alleles and genotypes among the cases and controls.

Group	Allele frequency*		Genotype frequency*			Hardy-Weinberg equilibrium (*p*-value)
	G	A	Wild Type (G/G)	Heterozygous(G/A)	Mutant (A/A)	
**Cases**	273 (46)	327 (55)	73 (24)	127 (42)	100 (33)	0.011
**Controls**	329 (55)	271 (45)	97 (32)	135 (45)	68 (23)	0.130

* Values in parenthesis are percentages.

### The association of the variant to the development of MDD

The association of the rs6265 SNP to the risk of MDD development was tested by performing logistic regression analysis. An association was considered significant based on the *p*-value and the odds ratio of the different inheritance models. In the present study, the significance value was obtained for the codominant, dominant, and recessive model (*p*<0.05) with the odds ratio of 1.95 (95% CI 1.27–3.01), 1.49 (95%CI 1.04–2.13), and 1.71 (95% CI 1.19–2.45), respectively. The selection of the best inheritance model was made by considering the lowest Akaike’s Information Criterion (AIC) value, which indicates a minimum discrepancy among the probability distribution and the true distribution. Based on the AIC value, the recessive model was the best inheritance model of *BDNF* rs6265 (*p* = 0.0035) (Table 7), indicating that two copies of the mutant allele are required to elevate the risk of developing MDD by 1.71 fold.

**Table 7:** The association of BDNF rs6265 to MDD (n = 599) with crude odds ratio of the different inheritance models.

Model	Genotype	Case	Control	OR (95%CI)	*p* value	AIC
**Codominant**	G/G	73 (24.3%)	97 (32.3%)	1.00	0.0075	828
	G/A	127 (42.3%)	135 (45%)	1.25 (0.85–1.84)		
	A/A	100 (33.3%)	68 (22.7%)	1.95 (1.27–3.01)		
**Dominant**	G/G	73 (24.3%)	97(32.3%)	1.00	0.03	831
	G/A-A/A	227 (75.7%)	203 (67.7%)	1.49 (1.04–2.13)		
**Recessive**	G/G-G/A	200 (66.7%)	232 (77.3%)	1.00	0.0035	827.3
	A/A	100 (33.3%)	68 (22.7%)	1.71 (1.19–2.45)		
**Overdominant**	G/G-A/A	173 (57.7%)	165 (55%)	1.00	0.51	835.3
	G/A	127(42.3%)	135 (45%)	0.90 (0.65–1.24)		

Due to the multifactorial nature of major depressive disorder, multivariable conditional logistic regression was conducted to estimate adjusted odds ratios of potential predictors of MDD, adjusting for potential confounders of age, gender, ethnicity, religion, job, income, education level, alcohol consumption, family history of neuropsychiatric disorders, depression, anxiety, schizophrenia, stroke, chronic disease, heart disease, diabetes, hypertension, arthritis, cancer and asthma. Table 8 lists the result for the conditional logistic regression of the association of the rs6265 SNP to the risk of MDD development following adjustment for all the above co-variates. Significance was obtained for the codominant, dominant, and recessive model (*p*<0.05) with the odds ratio of 2.49 (95% CI 1.33–4.65), 1.82 (95% CI 1.00–3.32), and 2.05 (95%CI 1.48–3.65), respectively. Upon the adjustment, the recessive model was the best model of inheritance of *BDNF* rs6265 indicating that having two copies of the recessive allele increase the risk of developing MDD by 2.05 folds when compared to the general population (*p = 0*.*015)*.

**Table 8:** The association of BDNF rs6265 to MDD (n = 598) upon the adjustment of odds ratio.

Model	Genotype	Case	Control	OR (95%CI)	*p* value	AIC
**Codominant**	G/G	73 (24.4%)	97 (32.3%)	1.00		732.5
	G/A	126 (42.1%)	135 (45%)	1.34(0.76–2.35)	0.31	
	A/A	100 (33.4%)	68 (22.7%)	2.49 (1.33–4.65)	0.004	
**Dominant**	G/G	73 (24.4%)	97 (32.3%)	1.00		734.9
	G/A-A/A	226 (75.6%)	203 (67.7%)	1.82 (1.00–3.32)	0.05	
**Recessive**	G/G-G/A	199 (66.6%)	232 (77.3%)	1.00		732.2
	A/A	100 (33.4%)	68 (22.7%)	2.05 (1.48–3.65)	0.015	
**Overdominant**	G/G-A/A	173 (57.9%)	165 (55%)	1.00		740.4
	G/A	126 (42.1%)	135 (45%)	0.86 (0.51–1.45)	0.57	

### Measurement of BDNF level using ELISA

**Fig 2:**
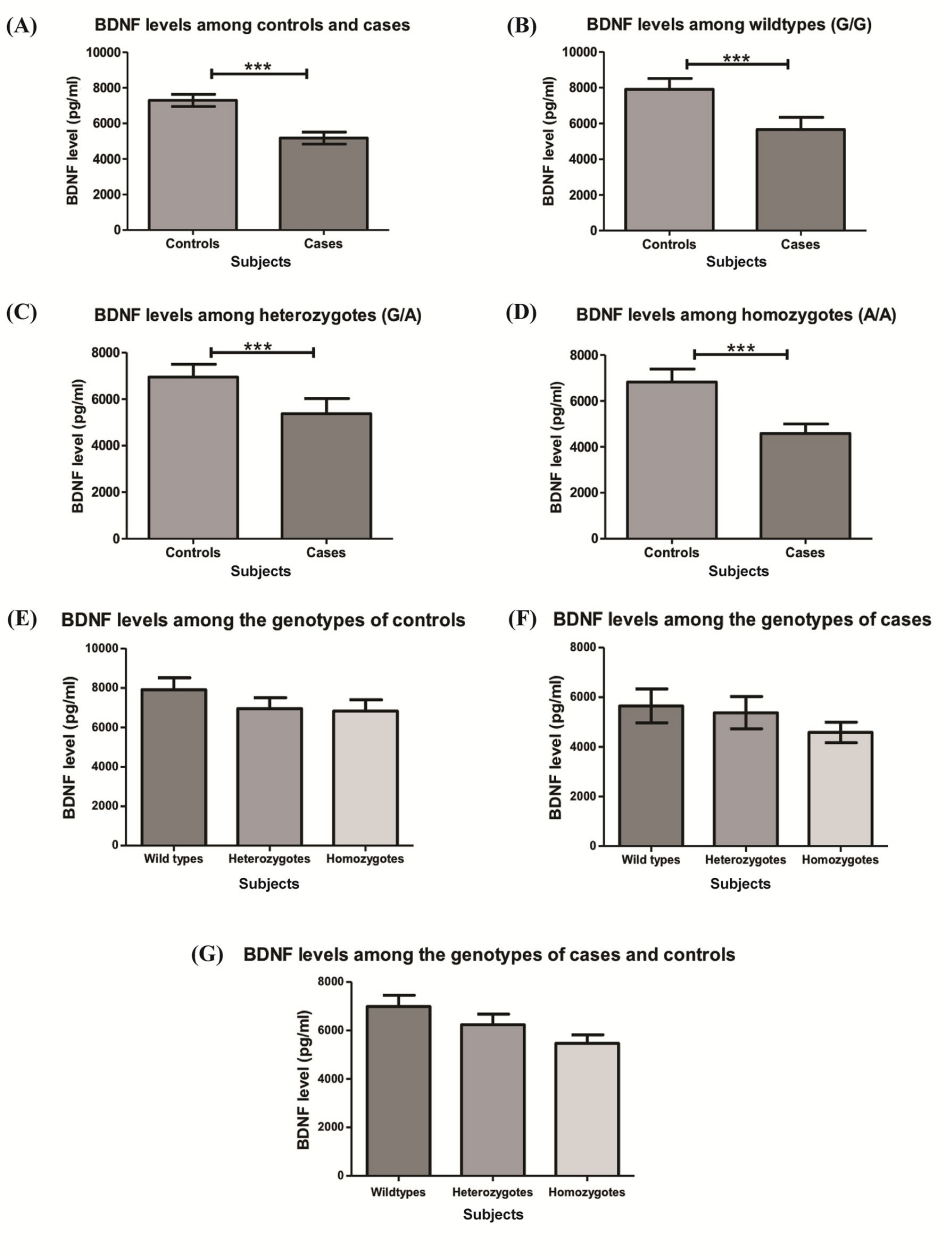
Measurement of plasma BDNF level. (A) The plasma BDNF level between the overall controls and cases (B) among subjects with wild type G/G, (C) heterozygous G/A and (D) homozygous A/A genotypes. The plasma BDNF level according to the different genotypes among (E) controls or (F) cases. (G) The plasma BDNF levels among the three genotypes regardless of subject groups (cases and controls). Results are represented as mean±SEM.

A total of 206 pairs of matched cases and controls were randomly selected for the Enzyme-linked immunosorbent assay analysis. The mean plasma BDNF level of the controls (7287±342.1 pg/ml) was significantly (*p*<0.0001) higher than the cases (5168±339.9 pg/ml) (Fig 2A). Also, a significant difference was obtained when comparing each of the individual genotypes of the controls against the genotypes of the cases using Wilcoxon signed rank test. A significant difference among the wild type of controls vs the wild type of the cases (*p* = 0.0002) with a mean plasma BDNF level of 7908±606.7 pg/ml vs 5652±681.9 pg/ml (Fig 2B). A similar pattern was also observed when comparing the BDNF level in the heterozygous genotypes of the controls against the cases (6947±555.4 pg/ml vs 5378±650 pg/ml, *p*<0.0001) (Fig 2C) and the BDNF level of mutants (6825±566.8 pg/ml vs 4582± 414.6 pg/ml, *p*<0.0001) (Fig 2D).

Following the difference in the BDNF level, the effect of the three genotypes on cases and controls was measured separately using Kruskal-Wallis test. There was no significant difference among the genotypes of the controls (G/G: 7908±606.7 pg/ml vs G/A: 6947±555.4 pg/ml vs A/A:6825±566.8 pg/ml, *p* = 0.5827) (Fig 2E) and among the genotypes of the cases (G/G: 5652±681.9 pg/ml vs G/A: 5378±650 pg/ml vs A/A: 4582±414.6 pg/ml, *p* = 0.5146) (Fig 2F). Finally, the effect of genotypes on the reduction of the BDNF level was studied regardless of the status (control vs cases) using Kruskal-Wallis test for nonparametric data and there was no significant difference between the three genotypes and the BDNF level (G/G: 6992±462.9 pg/ml vs G/A: 6236±439.7 pg/ml vs A/A: 5472±349.5 pg/ml; *p* = 0.0743) (Fig 2G). The mean BDNF levels, however, were consistently lower in subjects with heterozygous or mutant genotypes.

## Discussion

The brain derived neurotrophic factor is one of the most abundant neurotrophins in the mammalian brain and is involved in the function, development and survival of neurons [30]. The role of a single nucleotide polymorphism in the coding region of *BDNF* gene, *BDNF* rs6265 has been investigated in several neuropsychiatric disorders including major depressive disorder [16,31–34]. To date, there has been no study associating the *BDNF* rs6265 to MDD in the Malaysian population. In this study, we found that having the mutant variant increases the risk of MDD by approximately 2.05 (95%CI 1.48–3.65; *p* = 0.015) as compared to the wild type allele. Findings from our study agrees with the study by Ribeiro et al., (2007) [16] on the Caucasian population whereby they found a significant difference of the alleles between cases and controls (*p* = 0.005). The same study demonstrated that subjects with the mutant genotype had an odds ratio of 1.7 (95% CI 1.17–2.47) of developing MDD as compared to subjects of other genotypes. Similarly, Schumacher et al., (2005) studied two SNPs (rs988748 and rs6265) and a dinucleotide repeat (GT)_*n*_ of *BDNF* and found no association between the individual SNPs and depression [12]. However, an association was obtained at the haplotype level of both the SNPs and the dinucleotide repeat (*p* = 0.0057).

Coming closer to the Malaysian population, studies on elderly Taiwanese population showed that the distribution of A allele was higher in the cases as compared to controls (*p* = 0.001). Also, the study obtained an odds ratio of 2.49 (95% CI 1.40–4.46) indicating a 2.49fold increased risk of those bearing the mutant A/A genotype to develop MDD as compared to subjects with the G/G and G/A genotype [15]. Findings by Iga and colleagues (2007) on the Japanese population, found no effect of the variant on the MDD development but they found a significant association of the variant to clinical features of MDD such as suicide (*p* = 0.021) and psychosis (*p*<0.001) [14].

On the other hand, our result contradicts the findings from other case control studies on the Korean, Chinese, and Belgian populations that did not establish a significant association between the variant and MDD [13,32,35]. Likewise, no association has been obtained between the Val66Met polymorphism and MDD in a retrospective study on 7,389 Britain study subjects participating in the European Prospective Investigation into Cancer and Nutrition in Norfolk [36]. The reason for not obtaining significance in the above studies may be due to the small sample size used by some of these studies, difference in the age groups among cases and controls and usage of self-assessment methods that does not fully characterize the MDD phenotype.

In our study, the distribution of the alleles and genotypes for the controls was in Hardy-Weinberg equilibrium indicating that the allele and genotype frequency might remain constant if the conditions of Hardy-Weinberg Equilibrium were met. However, it was in Hardy-Weinberg disequilibrium for the cases. This may be because our cases were not randomly selected but were selected according to the disease. The obtained Val allele frequency concurs with the allele frequency of the Chinese (48.8%) [13], Japanese (55.8%) [14], Korean (45.8%) [32], and Taiwanese population (46.8%) [15] but was lower than the frequency observed in the Belgian population (82%) [35] and American Mexican population (89%) [16]. The Met allele frequency was also in agreement with the Asian population; Japanese (44.2%), Chinese (51.2%), Korean (54.2%) and Taiwanese (53.2%) but was higher than the Belgian (18%) and American Mexicans (11%). It is interesting to note that although some of the controls had the mutant genotype, yet, they did not suffer from MDD. MDD is a multifactorial disorder and the genetics is only one of the factors exerting the additive effect leading to the disorder [37]. In addition, those controls might have developed MDD at later life and could have been detected in a cohort study.

In this study, two other polymorphisms, namely, *BDNF* rs1048218 and rs1048220, were also screened. However, both polymorphisms were monomorphic throughout the initial screening involving 245 matched case-control subjects, thus were not analysed further. The observed monomorphic nature of the SNPs might be because the SNPs are rare and require a larger sample size to observe the effect. Also, these SNPs might be ethnic-specific as an association between the rs1048218 allele and early onset Alzheimer’s disorder was reported among Italian subjects (*P* = 0.047) [26].

Reduction in the BDNF level among MDD patients has been reported in a number of studies. The first study on the reduction of BDNF level in 30 MDD cases as compared to 30 controls was performed by Karege and colleagues (2002) [5]. They found a lower BDNF level (~15% reduction) in the serum of MDD cases as compared to controls (22.6±3 ng/ml and 26.5±7 ng/ml, *p*<0.01). Subsequently, several studies measuring the BDNF level in the plasma and serum of MDD patients reported similar findings [24,38–40]. Also, a meta-analysis study involving the findings from 10 case-control studies and 13 clinical trial studies, assessing 1,504 subjects reported a reduction in the plasma and serum BDNF level of MDD patients as compared to controls [41]. In addition, this study demonstrated an elevation in the BDNF levels among subjects following a treatment with antidepressants as compared to pre-treatment levels.

In the present study, the plasma BDNF was measured to reflect the level of BDNF in the brain. Previous studies have reported that BDNF can cross the blood brain barrier (BBB) [42] and a similar pattern has been observed in the plasma and brain BDNF levels upon maturation and aging in rats [5]. In addition, a study by Klein et al., 2011 [43], the levels of BDNF in the plasma, serum, blood and brain tissue of rat, mouse and pig were examined using ELISA. Findings from the study revealed a positive significant correlation between the BDNF levels of the whole blood and hippocampus (r^2^ = 0.44, *p* = 0.025) and between the BDNF levels in the plasma and hippocampus in pigs (r^2^ = 0.41, *p* = 0.025). These findings suggest that the level of BDNF in the plasma may reflect the level in the brain and thus may be used as an indicator for the severity of MDD. The result from ELISA of the current study displayed a significant difference between the BDNF level in the plasma of the controls as compared to the cases (control: 7287±4910 pg/ml vs cases: 5168 ±4879 pg/ml; *p*<0.0001). This is supported by the findings from other studies that also displayed higher BDNF level among the controls as compared to cases [24,39,40,44].

In this study, there was no significant difference in the BDNF level among the 3 genotypes of the cases (*p* = 0.5146) and the 3 genotypes of the controls (*p* = 0.5827). Also, no significant difference was observed among all the genotypes (Wild type G/G, Heterozygous G/A, Recessive A/A) regardless of the status (cases and controls) (*p* = 0.0743). Despite the lack of a significant difference, a higher BDNF level was observed among the wild type G/G carriers followed by the heterozygous G/A carriers with the least level observed among the mutant A/A carriers. On the other hand, a study by Colle and colleagues (2017) measured the BDNF level in 328 adult Caucasian MDD patients and found a significant difference in the plasma BDNF level among the genotypes (G/G: 1525.9±1183.3 pg/ml vs G/A: 1248.7±1081.8 pg/ml vs A/A: 1004.9±952.8 pg/ml, *p* = 0.04) [45].

There are several postulations to the above findings; firstly, the ELISA kit used in this study was not sensitive to mature BDNF alone but, it also detected pro-BDNF [46–47]. The effect of the *BDNF* variant on the level of mature BDNF (the functional form of the factor) was not determined and therefore may have been construed by the presence of pro-BDNF. Secondly, the reduction in the BDNF level in the cases as compared to the controls was not solely affected by rs6265 polymorphism but also influenced by other SNPs not screened in the study. Jin and colleagues conducted a study on Chinese population to determine the association of genetic variants of *BDNF* (rs10767664, rs1041635, rs962369, rs12273539, rs11030121 and rs4923468) to the moderate and severe allergic rhinitis. The study revealed a perfect linkage between *BDNF* rs10767664 and *BDNF* rs6265 and subjects with the rs10767664 A/A genotype were shown to be more susceptible to moderate-severe allergic rhinitis and had an increased plasma BDNF protein level [48]. Thirdly, the difference in the BDNF level might be due to epigenetic factors rather than due to genetic factors alone. Considering the aforementioned background, the assessment of the white blood cells of patients with MDD to measure the gene expression of 11 acetone deacetylase showed that in MDD, a significant increase in the expression of histone deacetylase 2 and 5 was observed in the depressed cases as compared to the healthy controls (HDAC2 *p*< 0.001and HDAC5 *p* = 0.001) [49]. Also, a study by Chagnon et al., 2015 on 19 women with MDD and anxiety disorder and 24 healthy controls revealed a higher DNA methylation in the BDNF gene among MDD/anxiety subjects as compared to healthy controls (mean ± SD: 2.92 ± 0.74 vs. 2.34 ± 0.42; *p* = 0.0026) [50], and finally, the change in the BDNF level was due to the effect of other environmental factors such as nutritional status [51–53], presence of co-morbidity such as diabetes mellitus [54], or due to the level of physical activity [55,56]. In addition, the disease severity and pharmacotherapy interventions may have affected the BDNF levels in the subjects as the BDNF level was shown to be affected by the administration of antidepressants and the given dose [57].

## Conclusion

The current study is the first to screen for *BDNF* rs6265 in the Malaysian population and associate it to the risk of development of MDD. Findings from this study indicate that having *BDNF* rs6265 increase the odds of developing MDD in the Malaysian population approximately by 2.05-fold. Hence, these findings strengthen the role of BDNF in the development of major depressive disorder in the Malaysian population and therefore serve a potential biomarker for early MDD screening in the future. Furthermore, a significant decrease in plasma BDNF levels was seen in the cases as compared to controls but not among the three genotypes. This implies a need for future studies to look at other single nucleotide polymorphisms in the *BDNF* gene and study their role and susceptibility to MDD both at the individual and at the haplotype level. In addition, further investigation of the cause and effect of reduction in the BDNF level is of equal importance.

## References

[1] American Psychiatric Association. Diagnostic and Statistical Manual of Mental Disorders. 2013.

[2] Depression and Other Common Mental Disorders: Global Health Estimates. Geneva: World Health Organization; 2017. Licence: CC BY-NC-SA 3.0 IGO.

[3] Vos T, Allen C, Arora M, Barber RM, Bhutta ZA, Brown A, et al Global, regional, and national incidence, prevalence, and years lived with disability for 310 diseases and injuries, 1990–2015: a systematic analysis for the Global Burden of Disease Study 2015. The Lancet. 2016 10 8;388(10053):1545–602. 10.1016/S0140-6736(16)31678-6 PMC5055577 27733282

[4] Institute for Public Health (IPH) 2015. National Health and Morbidity Survey 2015 (NHMS 2015) Vol. II: Non-Communicable Diseases, Risk Factors & Other Health Problems; 2015.

[5] Karege F, Schwald M, Cisse M. Postnatal developmental profile of brain-derived neurotrophic factor in rat brain and platelets. Neuroscience letters. 2002 8 16;328(3):261–4. 12147321 10.1016/s0304-3940(02)00529-3

[6] Duman RS, Monteggia LM. A neurotrophic model for stress-related mood disorders. Biological psychiatry. 2006 6 15;59(12):1116–27. 10.1016/j.biopsych.2006.02.013 16631126

[7] Lang UE, Borgwardt S. Molecular mechanisms of depression: perspectives on new treatment strategies. Cellular Physiology and Biochemistry. 2013;31(6):761–77. 10.1159/000350094 23735822

[8] Henderson CE. Role of neurotrophic factors in neuronal development. Current opinion in neurobiology. 1996 2 1;6(1):64–70. 8794045 10.1016/s0959-4388(96)80010-9

[9] Lewin GR, Barde YA. Physiology of the neurotrophins. Annual review of neuroscience. 1996 3;19(1):289–317. 10.1146/annurev.ne.19.030196.001445 8833445

[10] Bramham CR, Messaoudi E. BDNF function in adult synaptic plasticity: the synaptic consolidation hypothesis. Progress in neurobiology. 2005 6 1;76(2):99–125. 10.1016/j.pneurobio.2005.06.003 16099088

[11] Gottmann K, Mittmann T, Lessmann V. BDNF signaling in the formation, maturation and plasticity of glutamatergic and GABAergic synapses. Experimental brain research. 2009 12 1;199(3–4):203–34. 10.1007/s00221-009-1994-z 19777221

[12] Schumacher J, Jamra RA, Becker T, Ohlraun S, Klopp N, Binder EB, et al Evidence for a relationship between genetic variants at the brain-derived neurotrophic factor (BDNF) locus and major depression. Biological psychiatry. 2005 8 15;58(4):307–14. 10.1016/j.biopsych.2005.04.006 16005437

[13] Hong CJ, Huo SJ, Yen FC, Tung CL, Pan GM, Tsai SJ. Association study of a brain-derived neurotrophic-factor genetic polymorphism and mood disorders, age of onset and suicidal behavior. Neuropsychobiology. 2003;48(4):186–9. 10.1159/000074636 14673216

[14] Iga JI, Ueno SI, Yamauchi K, Numata S, Tayoshi‐Shibuya S, Kinouchi S, et al The Val66Met polymorphism of the brain‐derived neurotrophic factor gene is associated with psychotic feature and suicidal behavior in Japanese major depressive patients. American Journal of Medical Genetics Part B: Neuropsychiatric Genetics. 2007 12 5;144(8):1003–6. 10.1002/ajmg.b.30520 17510948

[15] Hwang JP, Tsai SJ, Hong CJ, Yang CH, Lirng JF, Yang YM. The Val66Met polymorphism of the brain-derived neurotrophic-factor gene is associated with geriatric depression. Neurobiology of aging. 2006 12 1;27(12):1834–7. 10.1016/j.neurobiolaging.2005.10.013 16343697

[16] Ribeiro L, Busnello JV, Cantor RM, Whelan F, Whittaker P, Deloukas P, et al The brain-derived neurotrophic factor rs6265 (Val66Met) polymorphism and depression in Mexican-Americans. Neuroreport. 2007 8 6;18(12):1291 10.1097/WNR.0b013e328273bcb0 17632285 PMC2686836

[17] Chen ZY, Patel PD, Sant G, Meng CX, Teng KK, Hempstead BL, et al Variant brain-derived neurotrophic factor (BDNF)(Met66) alters the intracellular trafficking and activity-dependent secretion of wild-type BDNF in neurosecretory cells and cortical neurons. Journal of Neuroscience. 2004 5 5;24(18):4401–11. 10.1523/JNEUROSCI.0348-04.2004 15128854 PMC6729450

[18] Chen ZY, Jing D, Bath KG, Ieraci A, Khan T, Siao CJ, et al Genetic variant BDNF (Val66Met) polymorphism alters anxiety-related behavior. Science. 2006 10 6;314(5796):140–3. 10.1126/science.1129663 17023662 PMC1880880

[19] Egan MF, Kojima M, Callicott JH, Goldberg TE, Kolachana BS, Bertolino A, et al The BDNF val66met polymorphism affects activity-dependent secretion of BDNF and human memory and hippocampal function. Cell. 2003 1 24;112(2):257–69. 12553913 10.1016/s0092-8674(03)00035-7

[20] Sen S, Duman R, Sanacora G. Serum brain-derived neurotrophic factor, depression, and antidepressant medications: meta-analyses and implications. Biological psychiatry. 2008 9 15;64(6):527–32. 10.1016/j.biopsych.2008.05.005 18571629 PMC2597158

[21] de Azevedo Cardoso T, Mondin TC, Wiener CD, Marques MB, de Avila Fucolo B, Pinheiro RT, et al Neurotrophic factors, clinical features and gender differences in depression. Neurochemical research. 2014 8 1;39(8):1571–8. 10.1007/s11064-014-1349-4 24899094

[22] Guilloux JP, Douillard-Guilloux G, Kota R, Wang X, Gardier AM, Martinowich K, et al Molecular evidence for BDNF-and GABA-related dysfunctions in the amygdala of female subjects with major depression. Molecular psychiatry. 2012 11;17(11):1130 10.1038/mp.2011.113 21912391 PMC3237836

[23] Tripp A, Oh H, Guilloux JP, Martinowich K, Lewis DA, Sibille E. Brain-derived neurotrophic factor signaling and subgenual anterior cingulate cortex dysfunction in major depressive disorder. American Journal of Psychiatry. 2012 11;169(11):1194–202. 10.1176/appi.ajp.2012.12020248 23128924 PMC3638149

[24] Karege F, Bondolfi G, Gervasoni N, Schwald M, Aubry JM, Bertschy G. Low brain-derived neurotrophic factor (BDNF) levels in serum of depressed patients probably results from lowered platelet BDNF release unrelated to platelet reactivity. Biological psychiatry. 2005 5 1;57(9):1068–72. 10.1016/j.biopsych.2005.01.008 15860348

[25] Banerjee R, Ghosh AK, Ghosh B, Bhattacharyya S, Mondal AC. Decreased mRNA and protein expression of BDNF, NGF, and their receptors in the hippocampus from suicide: an analysis in human postmortem brain. Clinical Medicine Insights: Pathology. 2013 1;6:CPath-S12530. 10.4137/CPath.S12530 PMC3767649 24031163

[26] Cozza A, Melissari E, Iacopetti P, Mariotti V, Tedde A, Nacmias B, et al SNPs in neurotrophin system genes and Alzheimer’s disease in an Italian population. Journal of Alzheimer’s Disease. 2008 1 1;15(1):61–70. 18780967 10.3233/jad-2008-15105

[27] Huang R, Huang J, Cathcart H, Smith S, Poduslo SE. Genetic variants in brain-derived neurotrophic factor associated with Alzheimer’s disease. Journal of medical genetics. 2007 2 1;44(2):e66-. 10.1136/jmg.2006.044883 17293537 PMC2598055

[28] Koshimizu H, Kiyosue K, Hara T, Hazama S, Suzuki S, Uegaki K, et al Multiple functions of precursor BDNF to CNS neurons: negative regulation of neurite growth, spine formation and cell survival. Molecular brain. 2009 12;2(1):27. 19674479 10.1186/1756-6606-2-27 PMC2743674

[29] Faris A, Yusof HH, Abidin SZ, Habib O, Cheah PS, Stanslas J, et al Development and Validation of High Resolution Melting Assays for High-Throughput Screening of BDNF rs6265 and DAT1 rs40184. Malaysian Journal of Medicine and Health Sciences. 2018; 14(SP1): 64–71.

[30] Dotta-Panichi RM, Bins HD, Tramontina JF, Ceresér KM, Aguiar BW, Paz AC, et al Serum concentrations of brain-derived neurotrophic factor and mental disorders in imprisoned women. Revista Brasileira de Psiquiatria. 2015 6;37(2):113–20. 10.1590/1516-4446-2014-1421 25714755

[31] Chen ZY, Bath K, McEwen B, Hempstead B, Lee F. Impact of genetic variant BDNF (Val66Met) on brain structure and function. InNovartis Foundation Symposium 2008 (Vol. 289, p. 180). NIH Public Access. 10.1002/9780470751251.ch14 PMC2735856 18497103

[32] Choi MJ, Kang RH, Lim SW, Oh KS, Lee MS. Brain-derived neurotrophic factor gene polymorphism (Val66Met) and citalopram response in major depressive disorder. Brain research. 2006 11 6;1118(1):176–82. 10.1016/j.brainres.2006.08.012 16979146

[33] Cole J, Weinberger DR, Mattay VS, Cheng X, Toga AW, Thompson PM, et al No effect of 5HTTLPR or BDNF Val66Met polymorphism on hippocampal morphology in major depression. Genes, Brain and Behavior. 2011 10;10(7):756–64. 10.1111/j.1601-183X.2011.00714.x PMC3420971 21692988

[34] Frodl T, Schüle C, Schmitt G, Born C, Baghai T, Zill P, et al Association of the brain-derived neurotrophic factor Val66Met polymorphism with reduced hippocampal volumes in major depression. Archives of General Psychiatry. 2007 4 1;64(4):410–6. 10.1001/archpsyc.64.4.410 17404118

[35] Oswald P, Del-Favero J, Massat I, Souery D, Claes S, Van Broeckhoven C, et al No implication of brain-derived neurotrophic factor (BDNF) gene in unipolar affective disorder: Evidence from Belgian first and replication patient–control studies. European neuropsychopharmacology. 2005 10 1;15(5):491–5. 10.1016/j.euroneuro.2005.01.001 16139165

[36] Surtees PG, Wainwright NW, Willis-Owen SA, Sandhu MS, Luben R, Day NE, et al No association between the BDNF Val66Met polymorphism and mood status in a non-clinical community sample of 7389 older adults. Journal of psychiatric research. 2007 8 1;41(5):404–9. 10.1016/j.jpsychires.2006.01.004 16497333

[37] Lopizzo N, Bocchio Chiavetto L, Cattane N, Plazzotta G, Tarazi FI, Pariante CM, et al Gene–environment interaction in major depression: focus on experience-dependent biological systems. Frontiers in psychiatry. 2015 5 8;6:68 10.3389/fpsyt.2015.00068 26005424 PMC4424810

[38] Aydemir C, Yalcin ES, Aksaray S, Kisa C, Yildirim SG, Uzbay T, et al Brain-derived neurotrophic factor (BDNF) changes in the serum of depressed women. Progress in Neuro-Psychopharmacology and Biological Psychiatry. 2006 9 30;30(7):1256–60. 10.1016/j.pnpbp.2006.03.025 16647794

[39] Lee BH, Kim H, Park SH, Kim YK. Decreased plasma BDNF level in depressive patients. Journal of affective disorders. 2007 8 1;101(1–3):239–44. 10.1016/j.jad.2006.11.005 17173978

[40] Piccinni A, Marazziti D, Catena M, Domenici L, Del Debbio A, Bianchi C, et al Plasma and serum brain-derived neurotrophic factor (BDNF) in depressed patients during 1 year of antidepressant treatments. Journal of affective disorders. 2008 1 1;105(1–3):279–83. 10.1016/j.jad.2007.05.005 17553570

[41] Brunoni AR, Lopes M, Fregni F. A systematic review and meta-analysis of clinical studies on major depression and BDNF levels: implications for the role of neuroplasticity in depression. International Journal of Neuropsychopharmacology. 2008 12 1;11(8):1169–80. 10.1017/S1461145708009309 18752720

[42] Pan W, Banks WA, Fasold MB, Bluth J, Kastin AJ. Transport of brain-derived neurotrophic factor across the blood–brain barrier. Neuropharmacology. 1998 12 1;37(12):1553–61. 9886678 10.1016/s0028-3908(98)00141-5

[43] Klein AB, Williamson R, Santini MA, Clemmensen C, Ettrup A, Rios M, et al Blood BDNF concentrations reflect brain-tissue BDNF levels across species. International Journal of Neuropsychopharmacology. 2011 4 1;14(3):347–53. 10.1017/S1461145710000738 20604989

[44] Bocchio-Chiavetto L, Bagnardi V, Zanardini R, Molteni R, Gabriela Nielsen M, Placentino A, et al Serum and plasma BDNF levels in major depression: a replication study and meta-analyses. The World Journal of Biological Psychiatry. 2010 9 1;11(6):763–73. 10.3109/15622971003611319 20334574

[45] Colle R, Trabado S, David DJ, Brailly-Tabard S, Hardy P, Falissard B, et al Plasma BDNF level in major depression: biomarker of the Val66Met BDNF polymorphism and of the clinical course in Met Carrier patients. Neuropsychobiology. 2017;75(1):39–45. 10.1159/000478862 28848102

[46] Yoshida T, Ishikawa M, Iyo M, Hashimoto K. Serum levels of mature brain-derived neurotrophic factor (BDNF) and its precursor proBDNF in healthy subjects. The Open Clinical Chemistry Journal. 2012 4 13;5(1).

[47] Polacchini A, Metelli G, Francavilla R, Baj G, Florean M, Mascaretti LG, et al A method for reproducible measurements of serum BDNF: comparison of the performance of six commercial assays. Scientific reports. 2015 12 10;5:17989 10.1038/srep17989 26656852 PMC4675070

[48] Jin P, Andiappan AK, Quek JM, Lee B, Au B, Sio YY, et al A functional brain-derived neurotrophic factor (BDNF) gene variant increases the risk of moderate-to-severe allergic rhinitis. Journal of Allergy and Clinical Immunology. 2015 6 1;135(6):1486–93. 10.1016/j.jaci.2014.12.1870 25649076

[49] Hobara T, Uchida S, Otsuki K, Matsubara T, Funato H, Matsuo K, et al Altered gene expression of histone deacetylases in mood disorder patients. Journal of psychiatric research. 2010 4 1;44(5):263–70. 10.1016/j.jpsychires.2009.08.015 19767015

[50] Chagnon YC, Potvin O, Hudon C, Préville M. DNA methylation and single nucleotide variants in the brain-derived neurotrophic factor (BDNF) and oxytocin receptor (OXTR) genes are associated with anxiety/depression in older women. Frontiers in genetics. 2015 6 30;6:230 10.3389/fgene.2015.00230 26175754 PMC4485183

[51] Molteni R, Barnard RJ, Ying Z, Roberts CK, Gomez-Pinilla F. A high-fat, refined sugar diet reduces hippocampal brain-derived neurotrophic factor, neuronal plasticity, and learning. Neuroscience. 2002 7 19;112(4):803–14. 12088740 10.1016/s0306-4522(02)00123-9

[52] Molteni R, Wu A, Vaynman S, Ying Z, Barnard RJ, Gomez-Pinilla F. Exercise reverses the harmful effects of consumption of a high-fat diet on synaptic and behavioral plasticity associated to the action of brain-derived neurotrophic factor. Neuroscience. 2004 1 1;123(2):429–40. 14698750 10.1016/j.neuroscience.2003.09.020

[53] Hou Y, Aboukhatwa MA, Lei DL, Manaye K, Khan I, Luo Y. Anti-depressant natural flavonols modulate BDNF and beta amyloid in neurons and hippocampus of double TgAD mice. Neuropharmacology. 2010 5 1;58(6):911–20. 10.1016/j.neuropharm.2009.11.002 19917299 PMC2838959

[54] Suwa M, Kishimoto H, Nofuji Y, Nakano H, Sasaki H, Radak Z, et al Serum brain-derived neurotrophic factor level is increased and associated with obesity in newly diagnosed female patients with type 2 diabetes mellitus. Metabolism. 2006 7 1;55(7):852–7. 10.1016/j.metabol.2006.02.012 16784955

[55] Goekint M, Heyman E, Roelands B, Njemini R, Bautmans I, Mets T, et al No influence of noradrenaline manipulation on acute exercise-induced increase of brain-derived neurotrophic factor. Medicine & Science in Sports & Exercise. 2008 11 1;40(11):1990–6. 18845978 10.1249/MSS.0b013e31817eee85

[56] Zoladz JA, Pilc A, Majerczak J, Grandys M, Zapart-Bukowska J, Duda K. Endurance training increases plasma brain-derived neurotrophic factor concentration in young healthy men. J Physiol Pharmacol. 2008 12 1;59(Suppl 7):119–32. 19258661

[57] Watanabe K, Hashimoto E, Ukai W, Ishii T, Yoshinaga T, Ono T, et al Effect of antidepressants on brain-derived neurotrophic factor (BDNF) release from platelets in the rats. Progress in Neuro-Psychopharmacology and Biological Psychiatry. 2010 12 1;34(8):1450–4. 10.1016/j.pnpbp.2010.07.036 20708057

